# Preoperative Dependent Functional Status Is Associated with Worse 30-Day Pulmonary Complications in Surgical Aortic Valve Replacement: An Insight from the American College of Surgeons National Surgical Quality Improvement Program Database From 2005-2021

**DOI:** 10.21470/1678-9741-2025-0461

**Published:** 2026-09-01

**Authors:** Renxi Li, Deyanira J. Prastein

**Affiliations:** 1 The George Washington University School of Medicine and Health Sciences, Washington, DC, United States of America; 2 Department of Surgery, The George Washington University Hospital, Washington, DC, United States of America

**Keywords:** Functional Status, Heart Valve Prosthesis Implantation, Aortic Stenosis, Pneumonia, Mechanical Ventilation

## Abstract

**Introduction:**

While current cardiac surgery risk scores do not account for preoperative functional status, it has been linked to adverse outcomes in transcatheter aortic valve replacement (TAVR). However, in surgical aortic valve replacement (SAVR), preoperative functional status remains underexplored.

**Objective:**

To examine the impact of preoperative functional status on 30-day SAVR outcomes to provide insights into clinical decision-making and postoperative care. **Methods:** Patients undergoing SAVR were identified from the American College of Surgeons National Surgical Quality Improvement Program database from 2005 to 2021. A 1:3 propensity-score matching was applied to patients with dependent (DFS) and independent (IFS) functional status. Thirty-day outcomes were assessed.

**Results:**

After propensity-score matching, DFS and IFS patients showed similar mortality (11.11% *vs.* 9.42%, *P* = 0.39) and major adverse cardiovascular events (9.91% *vs.* 9.11%, *P* = 0.66). Patients with DFS had higher risks of pulmonary complications (29.43% *vs.* 21.64%, *P* < 0.01) including pneumonia (13.21% *vs.* 9.21%, *P* = 0.04) and prolonged mechanical ventilation over 48 hours (25.83% *vs.* 15.42%, *P* < 0.01) but lower bleeding requiring transfusion (51.65% *vs.* 67.49%, *P* < 0.01). DFS patients were more likely to be discharged not to home (71.52% *vs.* 47.75%, *P* < 0.01) and had longer operation time (315.2 ± 130.5 *vs.* 293.5 ± 119.8 min, *P* < 0.01) and LOS (18.47 ± 14.30 *vs.* 15.07 ± 12.83 days, *P* < 0.01).

**Conclusion:**

While SAVR is relatively safe in eligible DFS patients considering its mortality and cardiac outcomes, it is imperative to emphasize preoperative functional status assessments and closely monitor postoperative pulmonary complications.

## INTRODUCTION

Aortic stenosis (AS) is a common condition, particularly among the older population. In individuals aged 75 years and above, the incidence of AS reaches 12.4%, with 3.4% presenting with severe AS that necessitates intervention^[1]^. The prevalence of AS is expected to be doubled by 2050 due to the aging global population^[2]^. While surgical aortic valve replacement (SAVR) used to be the principal treatment for AS^[3]^, transcatheter aortic valve replacement (TAVR) has gained traction as a minimally invasive alternative over the past decade, which is especially beneficial for patients deemed high-risk or unsuitable for traditional surgery^[4,5]^.

Functional status represents an individual's capacity to carry out daily activities, such as self-care, fulfilling typical physical, mental, and social roles, as well as maintaining independence at home or within the community^[6]^. Functional status was found to have a strong correlation with postoperative outcomes in cardiac surgeries and with long-term quality of life (QoL)^[7,8,9,10,11]^. For instance, MacPhedran et al. found that preoperative low functional status is associated with significantly higher risks of mortality and morbidity after elective cardiac surgery^[10]^. Wang et al found that in addition to higher mortality, patients with functional status had longer intensive care unit and hospital stays after cardiac surgery^[11]^.

The two most prevalent risk scores in cardiac surgery, the Society of Thoracic Surgeons (STS) score^[12]^ and the European System for Cardiac Operative Risk Evaluation (EuroSCORE) II^[13]^, do not account for functional status. However, functional status is notably implicated in aortic valve replacement (AVR). For instance, postoperative functional status has been identified as a measure of QoL following both TAVR and SAVR^[14,15]^. In TAVR, preoperative functional status has been linked to 30-day mortality in the STS/ American College of Cardiology Transcatheter Valve Therapy registry, and a clinical risk model incorporating functional status has been formulated to monitor TAVR outcomes^[16]^.

However, the influence of preoperative functional status on SAVR outcomes remains underexplored. Consequently, this study sought to examine the impact of preoperative functional status on 30-day SAVR outcomes, aiming to provide insights into clinical decision-making and postoperative care for those with dependent functional status (DFS).

## METHODS

### Data Source

The American College of Surgeons National Surgical Quality Improvement Program (ACS-NSQIP) database from 2005 to 2021 was used in this retrospective study. Patients who underwent SAVR were identified by Current Procedural Terminology codes 33400, 33401, 33402, 33403, 33404, 33405, 33406, 33407, 33408, 33409, 33410, 33411, and 33412. Functional status in the ACS-NSQIP database was determined within 30 days before the operation. Independent functional status (IFS) is characterized by an individual's ability to carry out daily activities without assistance from others, which also includes patients who, despite using prosthetics or other medical assistive devices, can function independently. Partially DFS describes individuals who can perform some daily tasks but need some assistance. In contrast, fully DFS refers to those who are entirely reliant on others for daily activities^[17]^. Patients with DFS (partially or fully) and IFS were stratified into two cohorts.

### Preoperative Variables

Demographics and baseline preoperative characteristics of the DFS and IFS cohorts were compared, as summarized in Table 1 and Table 2, respectively. Demographics included sex, age, as well as race and ethnicity.

**Table 1.** Comparing the demographics between the DFS and IFS cohorts before and after 1:3 propensity-score matching.

	DFS (n = 341)	Pre-match		Post-match	
		IFS	*P*-value	IFS	*P*-value
		(n = 10,099)		(n = 966)	
Sex (n, %)					
Male	206 (60.41%)	6662 (65.97%)	0.04	575 (59.52%)	0.80
Female	135 (39.59%)	3428 (33.94%)	0.03	391 (40.48%)	0.80
**Age (n, %)**					
Age < 55 years old	55 (16.13%)	1421 (14.07%)	0.30	149 (15.42%)	0.86
Age ≥ 55 < 65 years old	50 (14.66%)	2033 (20.13%)	0.01	141 (14.6%)	1.00
Age ≥ 65 < 75 years old	107 (31.38%)	3239 (32.07%)	0.81	287 (29.71%)	0.53
Age ≥ 75 < 85 years old	106 (31.09%)	2876 (28.48%)	0.30	321 (33.23%)	0.54
Age ≥ 85 years	23 (6.74%)	530 (5.25%)	0.22	68 (7.04%)	0.90
**Race and ethnicity (n, %)**					
Caucasian	250 (73.31%)	6434 (63.71%)	< 0.01	720 (74.53%)	0.77
African American	21 (6.16%)	370 (3.66%)	0.03	59 (6.11%)	0.90
Hispanic	51 (14.96%)	646 (6.4%)	< 0.01	147 (15.22%)	0.66
Asian American	2 (0.59%)	132 (1.31%)	0.33	3 (0.31%)	0.61
American Indian or Alaska Native	3 (0.88%)	31 (0.31%)	0.10	11 (1.14%)	1.00
Native Hawaiian or Pacific Islander	1 (0.29%)	17 (0.17%)	0.45	4 (0.41%)	1.00
Other races	56 (16.42%)	3023 (29.93%)	< 0.01	152 (15.73%)	0.73

DFS=dependent functional status; IFS=independent functional status

**Table 2.** Comparing the baseline characteristics between the DFS and IFS cohorts before and after 1:3 propensity-score matching.

	DFS (n = 341)	Pre-match		Post-match	
		IFS	*P*-value	IFS	*P*-value
		(n = 10,099)		(n = 966)	
Partially DFS	254 (74.49%)	0 (0%)	NA	0 (0%)	NA
Fully DFS	87 (25.51%)	0 (0%)	NA	0 (0%)	NA
BMI > 30 kg/m^2^	141 (41.35%)	4308 (42.66%)	0.66	400 (41.41%)	1.00
History of smoking	50 (14.66%)	1431 (14.17%)	0.81	149 (15.42%)	0.93
DM	137 (40.18%)	2537 (25.12%)	< 0.01	403 (41.72%)	0.52
Dyspnea	212 (62.17%)	4981 (49.32%)	< 0.01	587 (60.77%)	0.85
COPD	51 (14.96%)	806 (7.98%)	< 0.01	161 (16.67%)	0.55
CHF	169 (49.56%)	1703 (16.86%)	< 0.01	449 (46.48%)	0.45
Hypertension	274 (80.35%)	7394 (73.22%)	< 0.01	769 (79.61%)	0.87
AKI	9 (2.64%)	54 (0.53%)	< 0.01	22 (2.28%)	0.68
Dialysis	31 (9.09%)	223 (2.21%)	< 0.01	82 (8.49%)	0.91
Preoperative sepsis	69 (20.23%)	337 (3.34%)	< 0.01	165 (17.08%)	0.40
Disseminated cancer	2 (0.59%)	40 (0.4%)	0.40	6 (0.62%)	1.00
Infection	30 (8.8%)	107 (1.06%)	< 0.01	57 (5.9%)	0.24
Steroid use	21 (6.16%)	356 (3.53%)	0.02	54 (5.59%)	0.78
Weight loss	12 (3.52%)	90 (0.89%)	< 0.01	26 (2.69%)	0.57
Bleeding disorders	52 (15.25%)	673 (6.66%)	< 0.01	145 (15.01%)	1.00
eGFR < 60 mL/min/1.73m^2^	158 (46.33%)	2916 (28.87%)	< 0.01	448 (46.38%)	0.85
Serum albumin < 3.4 g/dL	135 (39.59%)	1100 (10.89%)	< 0.01	341 (35.3%)	0.35
WBC > 11,000 counts/mL	74 (21.7%)	714 (7.07%)	< 0.01	191 (19.77%)	0.75
Hematocrit < 37%	238 (69.79%)	3111 (30.81%)	< 0.01	666 (68.94%)	1.00
Platelet < 150,000 counts/mL	65 (19.06%)	1499 (14.84%)	0.04	169 (17.49%)	0.68
BUN > 23 mg/dL	143 (41.94%)	2640 (26.14%)	< 0.01	404 (41.82%)	1.00
PTT > 60 seconds	34 (9.97%)	247 (2.45%)	< 0.01	76 (7.87%)	0.42
PT > 30 seconds	3 (0.88%)	8 (0.08%)	< 0.01	5 (0.52%)	1.00
INR > 2	77 (22.58%)	4348 (43.05%)	< 0.01	247 (25.57%)	0.42
ASA score of 4 or 5	300 (87.98%)	8044 (79.65%)	< 0.01	846 (87.58%)	1.00
Emergency presentation	43 (12.61%)	475 (4.7%)	< 0.01	102 (10.56%)	0.68

AKI=acute kidney injury; ASA=American Society of Anesthesiology; BMI=body mass index; BUN=blood urea nitrogen; CHF=congestive heart failure; COPD=chronic obstructive pulmonary disease; DFS=dependent functional status; DM=diabetes mellitus; eGFR=estimated glomerular filtration rate; IFS=independent functional status; INR=international normalized ratio; NA=not applicable; PT=prothrombin time; PTT=partial thromboplastin time; WBC=white blood cells

### Perioperative Outcomes

Thirty-day postoperative outcomes assessed following SAVR included mortality, major adverse cardiovascular events (MACE), cardiac complications, stroke, pulmonary complications, renal complications, sepsis, venous thromboembolism, wound complications, and bleeding requiring transfusion. In addition, unplanned reoperation, discharge not to home, 30-day readmission, operation time, and length of stay (LOS) were compared. The major 30-day morbidities were reported as composite outcomes. Cardiac complications were defined as myocardial infarction and cardiac arrest requiring cardiopulmonary resuscitation. MACE included cardiac complications and stroke. Pulmonary complications consisted of pneumonia, unplanned reintubation, and prolonged mechanical ventilation over 48 hours. Renal complications included progressive renal insufficiency (rise in serum creatinine by > 2mg/dL compared to the preoperative value) and acute renal failure requiring renal replacement therapy. Wound complications included superficial and deep surgical site infections, organ space infections, and wound dehiscence.

### Statistical Analysis

All preoperative variables were compared by Fisher’s exact tests. To account for the preoperative differences and mismatched sample sizes between the DFS and IFS cohorts, a propensity-score matching was conducted in a 1:3 ratio (DFS: IFS) using a Greedy Matching Algorithm with a 2% caliper. After the propensity-score matching, Fisher's exact tests were used to compare binary postoperative outcomes, and two-tailed independent *t*-tests were used to compare continuous postoperative outcomes.

All statistical analyses were performed by SAS, version 9.4. A *P*-value < 0.05 was considered statistically significant. The ACS-NSQIP dataset was accessed from The George Washington University, where all subsequent statistical analyses were conducted. Since this retrospective study used the de-identified ACS-NSQIP dataset, an Institutional Review Board (IRB) review was exempted. The authors have full access to the dataset and are held fully responsible for the integrity of all statistical analyses.

## RESULTS

From 2005 to 2021, there were 341 patients with DFS (74.49% partially DFS and 25.51% fully DFS) and 10,099 patients with IFS identified from the ACS-NSQIP database. After 1:3 propensity-score matching, 966 IFS were matched with the DFS group.

A comparison of the demographics between the DFS and IFS cohorts before and after 1:3 propensity-score matching is shown in Table 1. Patients with DFS were more likely to be female (39.59% *vs.* 33.94%, *P* = 0.03), Caucasians (73.31% *vs.* 63.71%, *P* < 0.01), African Americans (6.16% *vs.* 3.66%, *P* = 0.03), and Hispanics (14.96% *vs.* 6.40%, *P* < 0.01), but less likely to be males (60.41% *vs.* 65.97%, *P* = 0.04), have age between 55 and 65 years (14.66% *vs.* 20.13%, *P* = 0.01), or be other races (16.42% *vs.* 29.93%, *P* < 0.01). All demographic differences were matched by the propensity-score matching.

The baseline characteristics between the DFS and IFS cohorts before and after 1:3 propensity-score matching are shown in Table 2. Compared to IFS patients, patients with DFS were more likely to have diabetes mellitus (40.18% *vs.* 25.12%, *P* < 0.01), dyspnea (62.17% *vs.* 49.32%, *P* < 0.01), chronic obstructive pulmonary disease (14.96% *vs.* 7.98%, *P* < 0.01), congestive heart failure (49.56% *vs.* 16.86%, *P* < 0.01), hypertension (80.35% *vs.* 73.22%, *P* < 0.01), acute kidney injury (2.64% *vs.* 0.53%, *P* < 0.01), dialysis (9.09% *vs.* 2.21%, *P* < 0.01), preoperative sepsis (20.23% *vs.* 3.34%, *P* < 0.01), infection (8.80% *vs.* 1.06%, *P* < 0.01), steroid use (6.16% *vs.* 3.53%, *P* = 0.02), weight loss (3.52% *vs.* 0.89%, *P* < 0.01), bleeding disorders (15.25% *vs.* 6.66%, *P* < 0.01), estimated glomerular filtration rate < 60 mL/min/1.73m^2^ (46.33% *vs.* 28.87%, *P* < 0.01), serum albumin < 3.4 g/L (39.59% *vs.* 10.89%, *P* < 0.01), white blood cells > 11,000 counts/mL (21.70% *vs.* 7.07%, *P* < 0.01), hematocrit < 37% (69.79% *vs.* 30.81%, *P* < 0.01), platelet < 150,000 counts/mL (19.06% *vs.* 14.84%, *P* = 0.04), blood urea nitrogen > 23 mg/dL (41.94% *vs.* 26.14%, *P* < 0.01), partial thromboplastin time > 60 seconds (9.97% *vs.* 2.45%, *P* < 0.01), prothrombin time > 30 seconds (0.88% *vs.* 0.08%, *P* < 0.01), American Society of Anesthesiology score of 4 or 5 (87.98% *vs.* 79.65%, *P* < 0.01), and emergency presentation (12.61% *vs.* 4.70%, *P* < 0.01) but less international normalized ratio > 2 (22.58% *vs.* 43.05%, *P* < 0.01). All differences in baseline characteristics were addressed by the propensity-score matching. Table 3 shows the comparison of the 30-day perioperative outcomes between the DFS and IFS cohorts after 1:3 propensity-score matching. Patients with DFS were at higher risks of pulmonary complications (29.43% *vs.* 21.64%, *P* < 0.01) including pneumonia (13.21% *vs.* 9.21%, *P* = 0.04) and prolonged mechanical ventilation over 48 hours (25.83% *vs.* 15.42%, *P* < 0.01). DFS patients were also more likely to be discharged not to home (71.52% *vs.* 47.75%, *P* < 0.01) but at lower risks of bleeding requiring transfusion (51.65% *vs.* 67.49%, *P* < 0.01). In addition, DFS patients had longer operation time (315.2 ± 130.5 *vs.* 293.5 ± 119.8 mins, *P* < 0.01) and LOS (18.47 ± 14.30 *vs.* 15.07 ± 12.83 days, *P* < 0.01).

**Table 3.** Comparing 30-day perioperative outcomes between the DFS and IFS cohorts after 1:3 propensity-score matching.

	DFS	IFS	*P*-value
	(n = 341)	(n = 966)	
Mortality	37 (11.11%)	91 (9.42%)	0.39
MACE	33 (9.91%)	88 (9.11%)	0.66
Cardiac complications	21 (6.31%)	56 (5.8%)	0.79
Stroke	13 (3.9%)	35 (3.62%)	0.87
Pulmonary complications	98 (29.43%)	209 (21.64%)	< 0.01
Pneumonia	44 (13.21%)	89 (9.21%)	0.04
Unplanned reintubation	30 (9.01%)	73 (7.56%)	0.41
Prolonged mechanical ventilation over 48 hours	86 (25.83%)	149 (15.42%)	< 0.01
Renal complications	29 (8.71%)	78 (8.07%)	0.73
Sepsis	42 (12.61%)	86 (8.9%)	0.06
VTE	16 (4.8%)	25 (2.59%)	0.07
Wound complications	15 (4.5%)	36 (3.73%)	0.52
Bleeding requiring transfusion	172 (51.65%)	652 (67.49%)	< 0.01
Unplanned reoperation	38 (11.41%)	123 (12.73%)	0.56
Discharge not to home	118 (71.52%)	372 (47.75%)	< 0.01
Readmission	17 (5.11%)	78 (8.07%)	0.09
	**Mean ± SD**	**Mean ± SD**	***P*-value**
Operation time (mins)	315.20 ± 130.50	293.50 ± 119.80	< 0.01
LOS (days)	18.47 ± 14.30	15.07 ± 12.83	< 0.01

DFS=dependent functional status; IFS=independent functional status; LOS=length of stay; MACE=major adverse cardiovascular events; SD=standard deviation; VTE=venous thromboembolism

## DISCUSSION

This study assessed the influence of preoperative functional status on 30-day outcomes post-SAVR. Following a 1:3 propensity-score match, DFS patients exhibited increased risks of pulmonary complications, including pneumonia and extended mechanical ventilation, yet had reduced instances of bleeding necessitating transfusion. Additionally, DFS patients had a higher likelihood of being discharged to destinations other than home, had longer operation times, and extended LOS.

Since its U.S. Food and Drug Administration approval in 2011, TAVR has witnessed a rapid shift from SAVR, becoming the predominant approach for treating AVR by 2019^[18]^. While TAVR offers numerous short-term advantages, making it ideal for high-risk and/or elderly patients, concerns about stent durability persist, and it has been linked to increased long-term mortality^[19]^. For example, a recent study demonstrated that for patients under 65 years old, five-year survival after SAVR was nearly twice that of TAVR, while the five-year incidence of heart failure readmission was more than 50% lower in the SAVR group^[20]^. Consequently, as of now, SAVR remains a valuable option for low-risk and/or younger patients due to its durability and superior long-term outcomes^[21]^. This underscores the importance of continued research in SAVR.

While preoperative functional status has been demonstrated to be associated with adverse outcomes in TAVR^[10,11,16]^, the effect of functional status on SAVR outcomes remains unclear. This inquiry becomes particularly intriguing as evolving patient selection criteria increasingly direct higher-risk patients toward TAVR. There is limited evidence suggesting that comorbidity correlates with functional status^[22]^. Thus, it remains unclear whether DFS should be regarded as an independent risk factor in the selection criteria of AVR.

After adjusting for demographics and comorbidities, patients with DFS and IFS exhibited similar rates of mortality and cardiac outcomes, suggesting that SAVR is comparably effective in DFS patients. However, for non-cardiac complications, patients with DFS experienced increased pulmonary complications, notably pneumonia, and prolonged mechanical ventilation. The higher pneumonia rate in DFS patients aligned with prior research indicating an association between DFS and postoperative pneumonia^[23]^. Postoperative pneumonia is a prevalent non-cardiac morbidity following cardiac surgery that can elevate mortality rates by up to ninefold and extend the LOS by two weeks^[24]^. In this study, DFS patients were more prone to extended mechanical ventilation, suggesting a potential link to ventilator-associated pneumonia, where previous studies have indicated that 35.2% of patients intubated for over 48 hours can develop this condition^[25]^. Indeed, prolonged ventilation is a significant complication of open-heart surgery and serves as a stronger predictor of mortality^[26,27,28,29]^. Therefore, assessing preoperative functional status can be implicated in postoperative pulmonary complication management. Despite higher pulmonary complications, patients with DFS exhibited a decreased risk of bleeding that necessitated transfusion. While the underlying reasons remain uncertain, it might be attributed to the limited mobility of DFS patients, potentially resulting in fewer incidents of bleeding from surgical sites.

### Limitations

This study acknowledges some limitations. Firstly, the ACS-NSQIP does not include certain details, such as the degree of stenosis nor allow the calculation of the STS score or EuroSCORE II. The ACS-NSQIP database does not record intraoperative factors, such as cross-clamping time, cardiopulmonary bypass duration, valve size/type, or ventilation strategy, which could be confounding variables that impact postoperative outcomes, including pulmonary complications. Additionally, patients with DFS, often being in poorer overall health, might be deemed unsuitable for open heart surgery and instead be directed towards TAVR or conservative treatments, introducing a potential selection bias. Furthermore, the ACS-NSQIP's follow-up period is restricted to 30 days post surgery, limiting the assessment of the impact of functional status on long-term SAVR outcomes and QoL. Another limitation is the hospitals’ selective participation in the ACS-NSQIP program, which might affect the study's broader generalizability. However, it is worth noting that ACS-NSQIP is a nationally recognized database with a comprehensive sample size that is tailored for surgical outcome evaluations, which can offer valuable perspectives on the influence of functional status on SAVR.

## CONCLUSION

In conclusion, this study examined the impact of preoperative functional status on the 30-day outcomes following SAVR. After propensity-score matching, DFS and IFS patients showed similar mortality and cardiac outcomes. However, DFS patients had higher risks of pulmonary complications including pneumonia and prolonged mechanical ventilation, but less bleeding requiring transfusion. Furthermore, DFS patients were more often discharged to facilities other than their homes, had longer surgical time, and had extended LOS. These findings indicate that while SAVR is relatively safe in eligible DFS patients, it is imperative to emphasize preoperative functional status assessments and closely monitor postoperative pulmonary complications.

## Data Availability

The authors declare that the data will be available upon reasonable request to the authors.
